# Socioeconomic status and risk for child psychopathology: Exploring gene-environment interaction in the presence of gene-environment correlation using extended families in the Norwegian Mother, Father and Child Birth Cohort Study

**DOI:** 10.1111/jcpp.13872

**Published:** 2023-08-12

**Authors:** Isabella Badini, Yasmin I. Ahmadzadeh, Daniel L. Wechsler, Torkild H. Lyngstad, Christopher Rayner, Espen M. Eilertsen, Helena M. S. Zavos, Eivind Ystrom, Tom A. McAdams

**Affiliations:** 1 Social, Genetic, & Developmental Psychiatry Centre, Institute of Psychiatry, Psychology and Neuroscience, King’s College London, London, United Kingdom; 2 Department of Sociology and Human Geography, University of Oslo, Oslo, Norway; 3 PROMENTA Research Center, Department of Psychology, University of Oslo, Oslo, Norway; 4 Department of Psychology, Institute of Psychiatry, Psychology and Neuroscience, King’s College London, London, United Kingdom; 5 Department of Mental Disorders, Norwegian Institute of Public Health, Oslo, Norway; 6 Centre for Fertility and Health, Norwegian Institute of Public Health, Oslo, Norway

**Keywords:** Emotional problems, Behavioural problems, Socioeconomic status, MoBa, Gene-environment interaction

## Abstract

**Background:**

Low socioeconomic status (SES) is associated with increased risk for emotional and behavioural problems among children. Evidence from twin studies has shown that family SES moderates genetic and environmental influences on child mental health. However, it is also known that SES is itself under genetic influence and previous gene-environment interaction (GxE) studies have not incorporated the potential genetic overlap between child mental health and family SES into GxE analyses. We applied a novel approach using extended family data to investigate the moderation of aetiological influences on child emotional and behavioural problems by parental socioeconomic status in the presence of modelled gene-environment correlation.

**Methods:**

The sample comprised >28,100 children in extended-family units drawn from the Norwegian Mother, Father and Child Cohort Study (MoBa). Mothers reported children’s emotional and behavioural symptoms. Parents’ income and educational attainment were obtained through linkage to administrative register data. Bivariate moderation Multiple-Children-of-Twins-and-Siblings (MCoTS) models were used to analyse relationships between offspring outcomes (emotional and behavioural symptom scores) and parental socioeconomic moderators (income rank and educational attainment).

**Results:**

The aetiology of child emotional symptoms was moderated by maternal and paternal educational attainment. Shared environmental influences on child emotional symptoms were greater at lower levels of parents’ education. The aetiology of child behavioural symptoms was moderated by maternal, but not paternal, socioeconomic factors. Genetic factors shared between maternal income and child behavioural symptoms were greater in families with lower levels maternal income. Nonshared environmental influences on child behavioural symptoms were greater in families with higher maternal income and education.

**Conclusions:**

Parental socioeconomic indicators moderated familial influences and non-shared environmental influences on child emotional and behavioural outcomes. Maternal SES and child mental health share aetiological overlap such that shared genetic influence was greater at the lower end of the socioeconomic distribution. Our findings collectively highlight the role that family socioeconomic factors play in shaping the origins of child emotional and behavioural problems.

## Introduction

Socioeconomic disadvantage is an important indicator of environmental adversity implicated in the development of mental health conditions (Costello et al., 2003; Glymour et al., 2014; Kinge et al., 2021; Maggi et al., 2010). A systematic review from 23 countries indicated that children from families with low socioeconomic status (SES) were two to three times more likely to develop emotional and behavioural problems than children from more socioeconomically advantaged families (Reiss, 2013). Low family SES is associated with disadvantages that may affect children’s mental health, such as material hardship, poor-quality housing conditions, social deprivation, and exposure to stressful life situations (Braveman & Gottlieb, 2014; Wilkinson & Marmot, Michael, 2003).

When studying the effects of family socioeconomic conditions on child development, researchers use a range of indices intended to capture access to social and material resources (Diemer et al., 2013; Wilkinson & Marmot, Michael, 2003). Typically, these indices comprise one or a combination of parental educational attainment, income, and occupational status (Diemer et al., 2013). Although these indicators are moderately correlated, each has been shown to measure distinctive aspects of the socioeconomic environment (Duncan & Magnuson, 2003; Geyer et al., 2006). For example, previous studies have shown that parental income and educational attainment have stronger associations with child mental health than other measures of family SES (Lansford et al., 2019; Reiss, 2013).

Besides correlating with child mental health, the socioeconomic environment has been shown to moderate the contribution of genetic and environmental influences on child mental health (Rutter et al., 2006). Twin studies of emotional and behavioural problems in children and adolescents have reported lower heritability and higher shared environmental influences (influences that make family members more similar to one another) in low SES families compared to high SES families (Burt et al., 2016, 2020; Hendriks et al., 2019; Middeldorp et al., 2014; Tuvblad et al., 2006). This is an example of gene-environment interaction (GxE), whereby genetic influences on child mental health interact with the socioeconomic environment (Eaves et al., 1977). The findings from these studies could be taken as support for the bioecological framework, which proposes that more advantageous environments allow for greater expression of genetic differences, while more disadvantageous environments suppress them (Bronfenbrenner & Ceci, 1994).

As well as moderating familial influences on child mental health, family SES has also been found to moderate non-shared environmental influences (influences that make family members different from one another) on child behavioural problems (Hendriks et al., 2019; Middeldorp et al., 2014; Tuvblad et al., 2006). Results are inconsistent on the direction of this moderation effect and depends on the indicators of family SES used (Hendriks et al., 2019; Middeldorp et al., 2014; Tuvblad et al., 2006). This suggests that different indicators of SES may exert different moderating effects on child outcomes (Duncan & Magnuson, 2003; Geyer et al., 2006).

A shortcoming of previous twin studies that have examined the moderation of aetiological influences by family SES is that they do not model genetic overlap between SES and child psychopathology (i.e., gene-environment correlation). This has largely been due to limitations in the data used: child twin data cannot be used to estimate the heritability of environments that are entirely shared by twins, rendering it impossible to estimate the heritability of family SES (Purcell, 2002; Rijsdijk & Sham, 2002). Research using genome-wide genotype data have however shown that indicators of family SES are heritable (Krapohl & Plomin, 2016; Trzaskowski et al., 2014). Studies have also found evidence for genetic overlap between parental socioeconomic factors and child mental health (Krapohl & Plomin, 2016; Torvik et al., 2020; Trzaskowski et al., 2014). This suggests that parents are a source of genetic risk for offspring mental health and the socioeconomic conditions children grow up in, which is evidence of passive gene-environment correlation (rGE) (Kendler & Baker, 2007; Plomin et al., 1977). Genetic influence in the relationship between SES and mental health does not preclude a causal relationship between family SES and child mental health, but it does mean that genetically informed approaches are required to gain insight into the likely mechanisms underlying their association.

Previous GxE twin studies of family SES and child psychopathology have regressed out the main effect of SES on child psychopathology before testing for GxE to account for inflation of test statistics due to rGE (Purcell, 2002; van der Sluis et al., 2012). As such, these studies specifically focus on whether family SES moderates the aetiological influences *unique* to child psychopathology. This does not allow for investigation of either the nature of the covariance between family SES and child psychopathology, nor the moderation of the variance in child psychopathology that is shared with family SES. This is an issue because exclusion of the common variance between them may limit our understanding of the mechanisms through which socioeconomic disadvantage influences the aetiology of child mental health outcomes.

The Multiple-Children-of-Twins-and-Siblings (MCoTS) design provides an alternative approach to examine the moderating effect of family SES on the aetiology child emotional and behavioural outcomes. The MCoTS design involves using datasets comprising related parents and their children (i.e., extended families) to partition intergenerational associations into genetic and environmental sources of (co)variation (McAdams et al., 2018). The inclusion of multiple types of relatives means that family SES can vary within extended family units. This information allows the aetiological structure of socioeconomic indicators to be calculated and thus the genetic (and environmental) covariance between parental SES and child psychopathology. The MCoTS model can therefore be adapted to test for gene-environment interaction in the presence of gene-environment correlation.

The aim of this study was to investigate whether aetiological influences on child emotional and behavioural problems vary as a function of family SES as indexed by parental income and educational attainment. We applied moderation (GxE) MCoTS models to a large population-cohort study of twins, siblings, and half-siblings, and their children in Norway. Linked population-wide, administrative register data was used to index parental income and educational attainment.

## Methods

### Sample

Data were drawn from The Norwegian Mother and Child Cohort Study (MoBa) (Magnus et al., 2016) and from national administrative registers provided by Statistics Norway. MoBa is a population-based pregnancy cohort study conducted by the Norwegian Institute of Public Health. Participants were recruited from all over Norway from 1999–2008. The women consented to participation in 41% of pregnancies. The cohort includes ~114,500 children, 95,200 mothers, and 75,00 fathers. Kinship between participants has been identified through linkage with pedigree and zygosity information from the Medical Birth Registry of Norway and the Norwegian Twin Registry, respectively (T. S. Nilsen et al., 2013). The current sample comprised extended family units, identified via pairs of siblings (twins, full-siblings, or half-siblings) in the parent generation. Extended family units were identified separately for sibling pairs of mothers or fathers and their children (i.e., units of maternal/paternal siblings and their children were modelled separately). Within each extended family unit, data were used from up to two parent siblings and two children per parent. Parents who do not have participating extended family members were included in analyses as nuclear family units if they had more than one child in the study. Phenotypic data were drawn from version 12 of the quality assured MoBa data files. The current study also uses national register data on parents’ income and educational attainment. The Norwegian system of personal identification numbers was used to link register data with MoBa data.

### Ethical considerations

The establishment of MoBa and initial data collection was based on a license from the Norwegian Data Protection Agency and approval from The Regional Committees for Medical and Health Research Ethics. The MoBa cohort is currently regulated by the Norwegian Health Registry Act. The current study was approved by The Regional Committees for Medical and Health Research Ethics.

### Measures

#### Outcomes: Offspring emotional and behavioural symptoms

We analysed two offspring outcomes: emotional symptom scores and behavioural symptom scores. Both were measured by maternal report when children were ages 1.5, 3, and 5 years old, using items from the Child Behaviour Checklist (CBCL) for preschool children (Achenbach, 1992). This scale consists of two subscales. The internalising sub-scale includes 13 items that measure emotional symptoms. The externalising sub-scale includes 11 items that measure behavioural symptoms. Mothers reported agreement for each item based on a three-point Likert scale: 1 = Not true; 2 = Somewhat true; 3 = Very/often true. We combined item-level scores across the three measurement waves to create composite mean scores for early-life emotional (Cronbach’s alpha = 0.74) and behavioural (Cronbach’s alpha = 0.84) symptoms.

#### Moderators: Parental socioeconomic factors

We analysed the effects of four parental socioeconomic variables as moderators of offspring outcomes. These were maternal income, maternal education, paternal income, and paternal education, which were extracted from national register data. Data on parents’ total income (the sum of income from work capital gains, and benefits received during the calendar year) and educational attainment from 2000 to 2013 were included, corresponding to when child outcomes were assessed in MoBa (when children were aged 1.5, 3 and 5 years old).

At each time-point, we created a measure of income rank that indicates an individual’s position in the distribution of incomes within a cohort-sex-year-group (e.g., 2005 income for females/males born in 1983). The income rank measure was scaled between 0 and 1, with higher values denoting a higher income rank within the reference group. Income rank at these three time-points was used to calculate average income rank across early childhood for each parent.

At each time-point, level of educational attainment was indexed in accordance with the Norwegian Standard Classification of Education, with values ranging from 1 (primary education) to 8 (doctoral-level education). Education-level at these three time-points was used to calculate average education-level across early childhood for each parent.

#### Covariates and outcome adjustments

Prior to model fitting, outcomes were regressed on the following covariates: parental age, child year of birth, number of births, and child sex. Residual scores were then log transformed to correct for positive skew and all variables were standardised prior to model fitting. Transformation of non-normal sum scores to normality improves false positive rates and reduces bias in parameter estimates (Murray et al., 2016). Moderator scores were not transformed as scaling of the predictor has minimal impact on estimates of interactions (Van Hulle & Rathouz, 2015).

### Statistical analysis

#### The Multiple-Children-of-Twins-and-Siblings (MCoTS) design

The Multiple-Children-of-Twins-and-Siblings (MCoTS) model is an adapted version of the standard Children-of-Twins design (McAdams et al., 2018) that includes twins, full siblings, and half siblings in the parent generation, and up to two children per parent in the offspring generation. Like the classical twin design (Jinks & Fulker, 1970), these models compare similarities among family members of different genetic relatedness to decompose observed variance and covariance into genetic and environmental components. Differential genetic similarity among related parents means that children of monozygotic (MZ) twins (who share 100% of their DNA) are more related to their parent’s co-twin and their cousins compared to children of dizygotic (DZ) twins and full siblings (who share 50% of their segregating genes). Comparing associations between different classes of relatives in such samples (e.g., correlations between uncle/aunt and niece/nephew) allows for intergenerational transmission effects to be partitioned into passive genetic transmission, passive shared environmental transmission, and direct phenotypic components (see Table 1).

The MCoTS model decomposes variance in the parent trait (e.g., SES indicators) into parent genetic (A1), shared environmental (C1), and nonshared environmental (E1) components, and variance in child traits (e.g., emotional, and behavioural problems) into child genetic (A2), shared environmental (C2), and nonshared environmental (E2) components (Figure S1). Variance explained by A1, C1 and E1 are unique to the parent generation and non-overlapping with variance explained by A2, C2 and E2 in the child generation. The intergenerational association between the parental and child trait is partitioned into genetic transmission (A1’), shared environmental effects (C1’; indexing environmental influences shared across the extended family) and residual phenotypic transmission (p; accounting for non-genetic effects shared between the parent and child, and effects of any sources of confounding unaccounted for; Figure S1).

#### Using the MCoTS design to investigate moderation of offspring outcomes by parental socioeconomic factors

**Figure 1.**
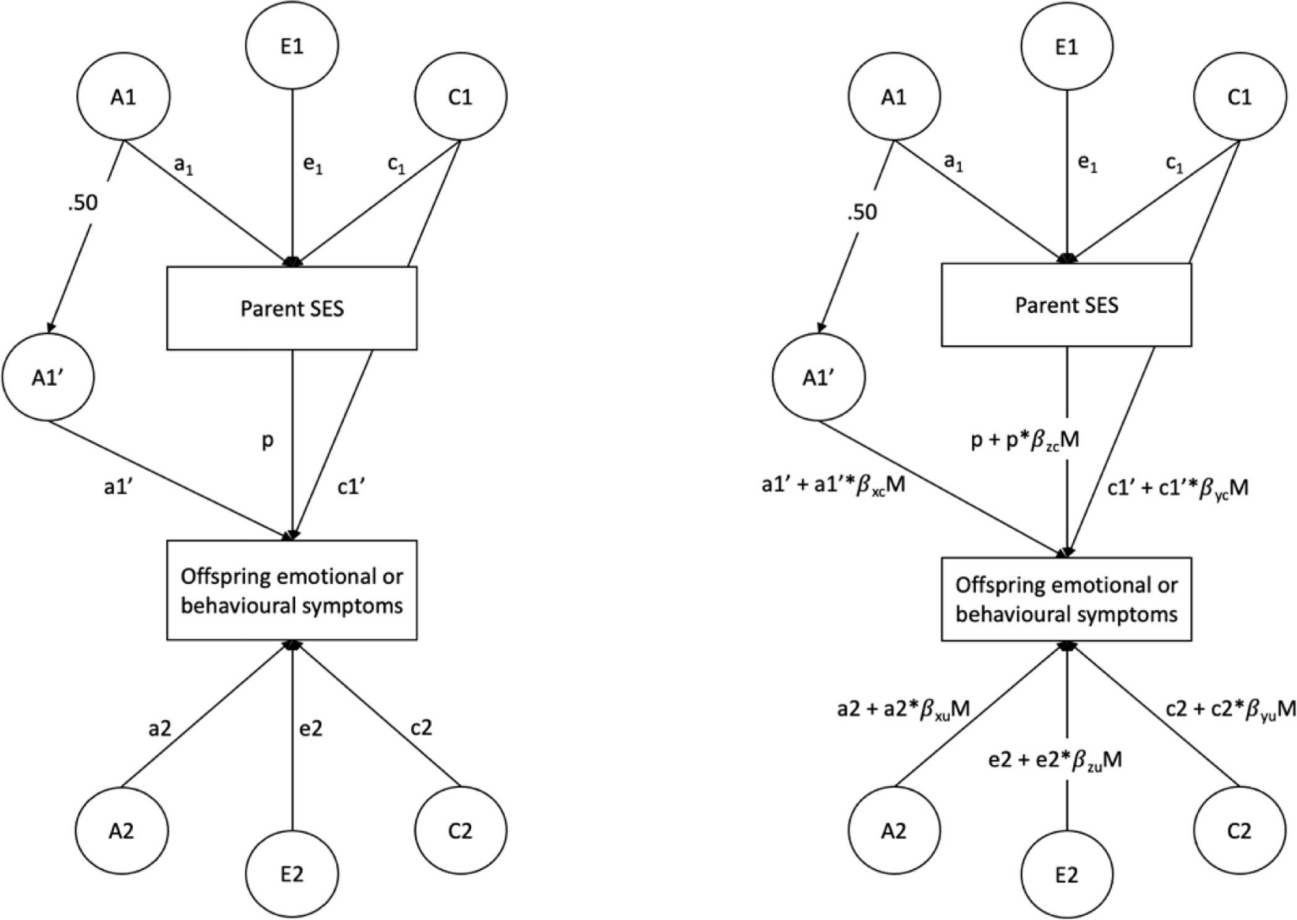
Partial path diagram of the Multiple-Children-of-Twins-and-Siblings (MCoTS) model showing the addition of moderation terms to the intergenerational and child trait paths (right) to the MCoTS model (left; Figure S1). The model is shown for only one sibling in the parent generation and one child. *Right figure.* A1 = additive genetic effects on parental trait; C1 = shared environmental effects on parental trait; E1 = nonshared environmental effects on parental trait; A1’ = genetic effects shared between parental trait and offspring trait; C1’ = extended family effects (i.e. shared environment of the parents influences offspring trait); A2 = genetic effects specific to offspring trait; C2 = shared environmental effects on offspring trait; E2 = nonshared environmental effects on offspring trait; p = residual phenotypic association after accounting for genetic and environmental overlap. *Left figure.* A1’, C1’, and p are the variance components common to parent SES (the moderator) and child emotional or behavioural symptoms. A2, C2, and E2 are the variance components unique to child emotional or behavioural symptoms. β coefficients index the direction and magnitude of moderation. The total variance of the trait can be calculated by squaring and summing all the paths leading to it: $\text{Var}(\text{T}|\text{M})=(\text{a}1’+\text{a}1’*{\text{β}}_{\text{xc}}\text{M}{)}^{2}+(\text{p}+\text{p}*{\text{β}}_{\text{zc}}\text{M}{)}^{2}+(\text{c}1’+\text{c}1’*{\text{β}}_{\text{yc}}\text{M}{)}^{2}+(\text{a}2+\text{a}2*{\text{β}}_{\text{xu}}\text{M}{)}^{2}+(\text{e}2+\text{e}2*{\text{β}}_{\text{zu}}\text{M}{)}^{2}+(\text{c}2+\text{c}2*{\text{β}}_{\text{yu}}\text{M}{)}^{2}$. *Note.* Var = variance; T = trait; M = moderator; The loadings of the cross-paths connecting M to T consist of parts unrelated to the moderator M, i.e., a1’, p, and c1’ and parts that depend on M via weights ${\text{β}}_{\text{xc}}$, ${\text{β}}_{\text{zc}}$, and ${\text{β}}_{\text{yc}}$. The loadings of the paths unique to T consist of parts that are unrelated to M, i.e., a2, e2 and c2, and parts that depend on M via weights ${\text{β}}_{\text{xu}}$, ${\text{β}}_{\text{zu}}$, and ${\text{β}}_{\text{yu}}$.

To investigate whether parental socioeconomic factors moderate the aetiology of child emotional and behavioural problems, we adapted the MCoTS model to include moderation terms on the intergenerational and child trait paths (Figure 1). This is based on the bivariate moderation model proposed by Purcell (2002) (Figure S2), in which it is possible to simultaneously model: 1) shared genetic and environmental effects between the moderator and trait; 2) the moderation of the genetic and environmental variance components shared between the moderator and trait; 3) the moderation of the variance components unique to the trait. The bivariate moderation model requires the moderator variable to vary within extended families, hence it has not previously been applied to studies of twin children who share the same nuclear family environment. Because SES varies within extended family units (i.e., across adult siblings who have children), the MCoTS design allows us to utilise the bivariate moderation model to estimate whether parental SES moderates the intergenerational and unique genetic and environmental influences on child psychopathology (Figure 1).

We conducted eight bivariate moderation MCoTS models to analyse the relationships between two offspring outcomes (emotional and behavioural symptom scores) and four parental socioeconomic moderators (income rank and educational attainment). To test for the significance of moderation effects, we compared each full moderation model with a no-moderation model, in which all the moderation parameters were dropped. Significance of individual variance components was indicated by 95% confidence intervals (CI) around the moderated parameters from the full model^1^. Models were fitted using full-information-maximum-likelihood and compared using the x^2^ distribution of the −2 log-likelihood model fit index and Akaike’s Information Criterion (AIC) (Akaike, 1987). Analyses were performed in R version 4.0.3 using the open source package OpenMx v.2.12.1 (Neale et al., 2016).

## Results

### Descriptive statistics

Table 1 presents an overview of the study sample. Mothers at recruitment were aged on average 30.22 (SD=4.18) years and fathers 32.04 (SD = 4.88). 51% of the children included were males. Phenotypic correlations between all study variables are presented in Table S1. Child emotional and behavioural symptom scores were moderately correlated (*r* = 0.39). Parental socioeconomic factors were negatively correlated with child emotional and behavioural scores (*r* ranged from −0.04 to −0.11). Transformed variables had approximately normal distributions (Figure S3).

### MCoTS moderation models

#### Moderation of offspring mental health outcomes by maternal socioeconomic factors

##### Overall model fit

Four moderation MCoTS models were applied to test whether the aetiology of child emotional and behavioural symptoms were moderated by two maternal exposures: maternal income and education attainment. Dropping all moderation parameters resulted in a significant worsening in model fit compared to the full moderation models (Table S2). Moderated parameter estimates from the full moderation model are presented in Table S3. Figures 3 and 4 show the unstandardised variance components for child emotional and behavioural problems as a function of maternal income and educational attainment.

##### Emotional symptoms

**Figure 2.**
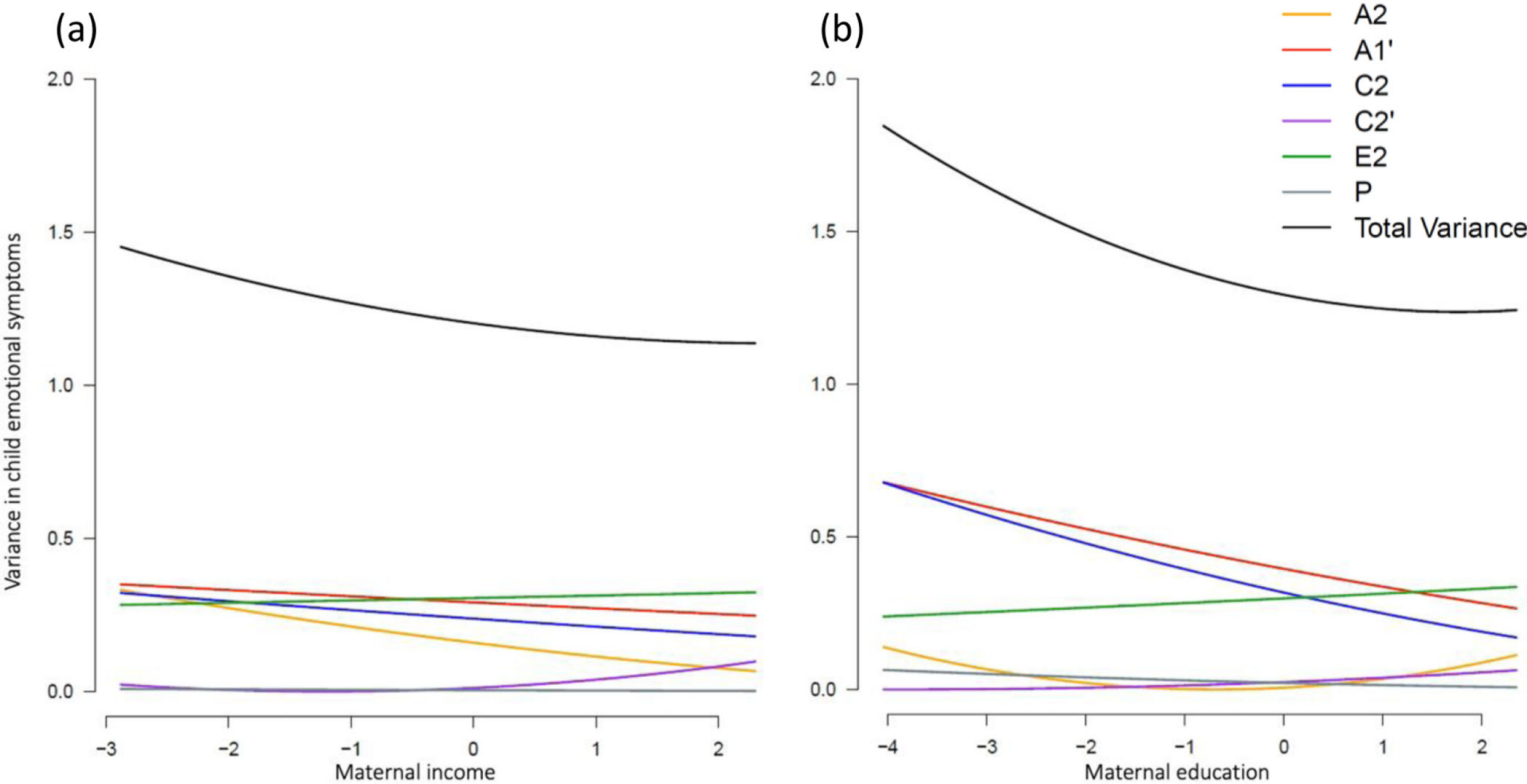
Unstandardised variance components in child emotional problems moderated by maternal income rank (a) and educational attainment (b).

Lower levels of maternal income and education were associated with greater variation in child emotional symptoms (Figure 2a and Figure 2b, respectively). Figure 2a suggests genetic influences on child emotional symptoms (A1’ and A2 variance components) and shared environmental influences unique to child emotional outcomes (C2 variance component) were greater at lower versus higher levels of maternal income. Although moderation terms could not all be dropped from the model without a significant loss of model fit (Table S2), no single variance component for emotional outcomes appeared significantly moderated by maternal income when confidence intervals around the moderation terms were inspected (see Table S3). Figure 2b suggests moderation effects on the A1’ and C2 variance components, such that genetic factors common to maternal education and child emotional outcomes (A1’), and shared environmental influences unique to child emotional outcomes (C2), were greater at lower levels of maternal education. Examination of the confidence intervals around the moderation parameter estimates from the full model showed significant moderation on the shared environmental component unique to child emotional outcomes (C2) as a function of maternal education (β_yu_ = −0.06, 95% CI [−0.10, −0.01]; Table S3).

##### Behavioural symptoms

**Figure 3.**
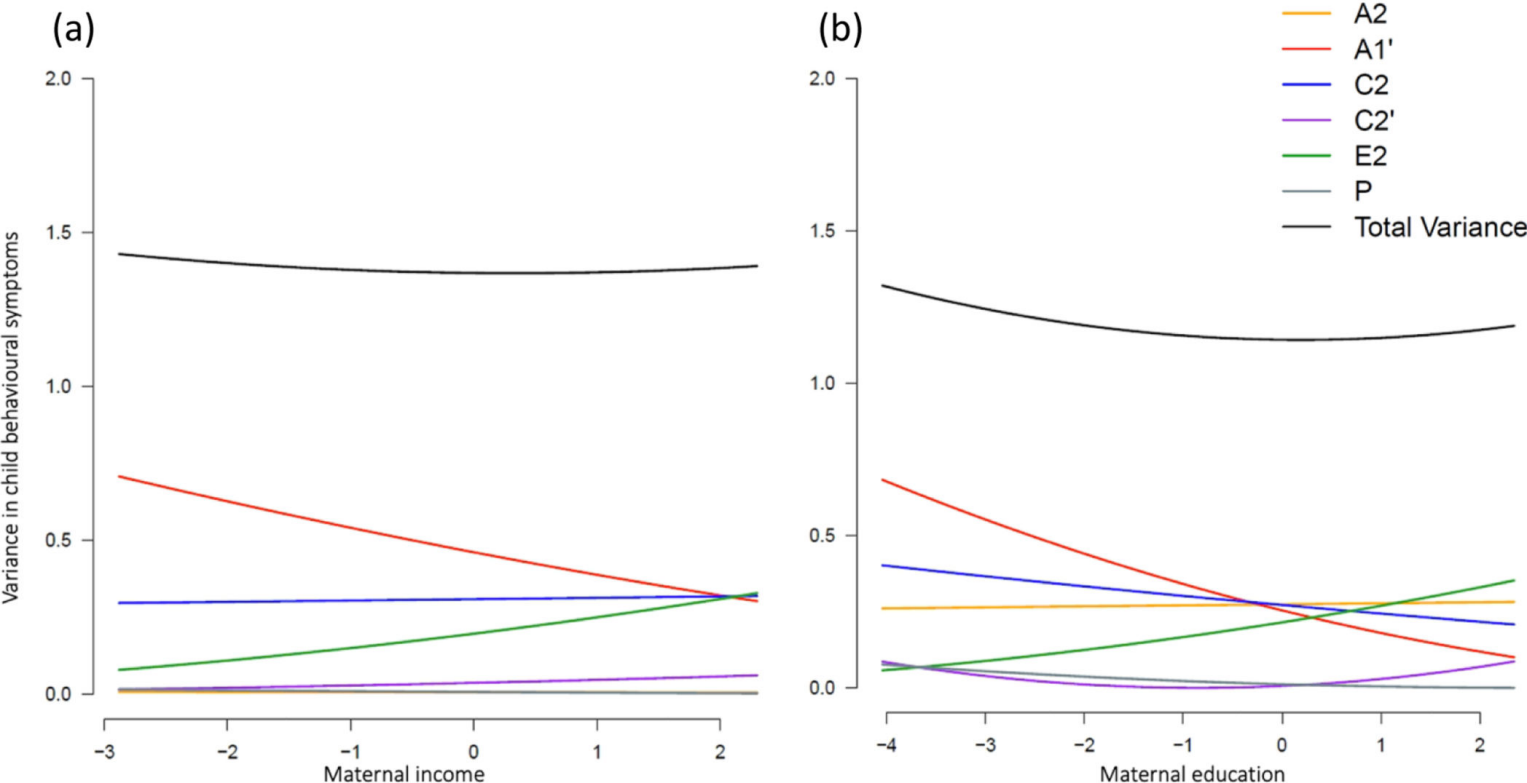
Unstandardised variance components in child behavioural problems moderated by maternal income rank (a) and education attainment (b).

Total phenotypic variation in child behavioural symptoms was stable across levels of maternal income (Figure 3a) and education (Figure 3b). Figure’s 3a and 3b suggest however that genetic influences common to maternal socioeconomic factors and child behavioural outcomes (A1’) were greater at lower levels of maternal income and education, whereas nonshared environmental factors (E2) showed a stronger influence on child behavioural outcomes with increasing maternal income and education. Examination of the confidence intervals around the moderation terms from the full model showed significant moderation on the shared genetic component (A1’; β_xc_ = 0.06, 95% CI [0.01, 0.09]) and the nonshared environmental component unique to child emotional outcomes (E2; β_zu_ = 0.06, 95% CI [0.02, 0.09]) as a function of maternal income (Table S3). Significant moderation was observed on the nonshared environmental component (E2) as a function of maternal education (β_zu_ = 0.06, 95% CI [0.02, 0.08]; Table S3).

#### Moderation of offspring mental health outcomes by paternal socioeconomic factors

##### Overall model fit

Four moderation MCoTS models were applied to test whether the aetiology of child emotional and behavioural symptoms were moderated by two paternal exposures: income and educational attainment. Dropping all moderation parameters resulted in a significant decrease in fit compared to the full models for child emotional and behavioural symptoms (Table S4). Moderated parameter estimates from the full moderation model are presented in Table S5.

##### Emotional symptoms

**Figure 4.**
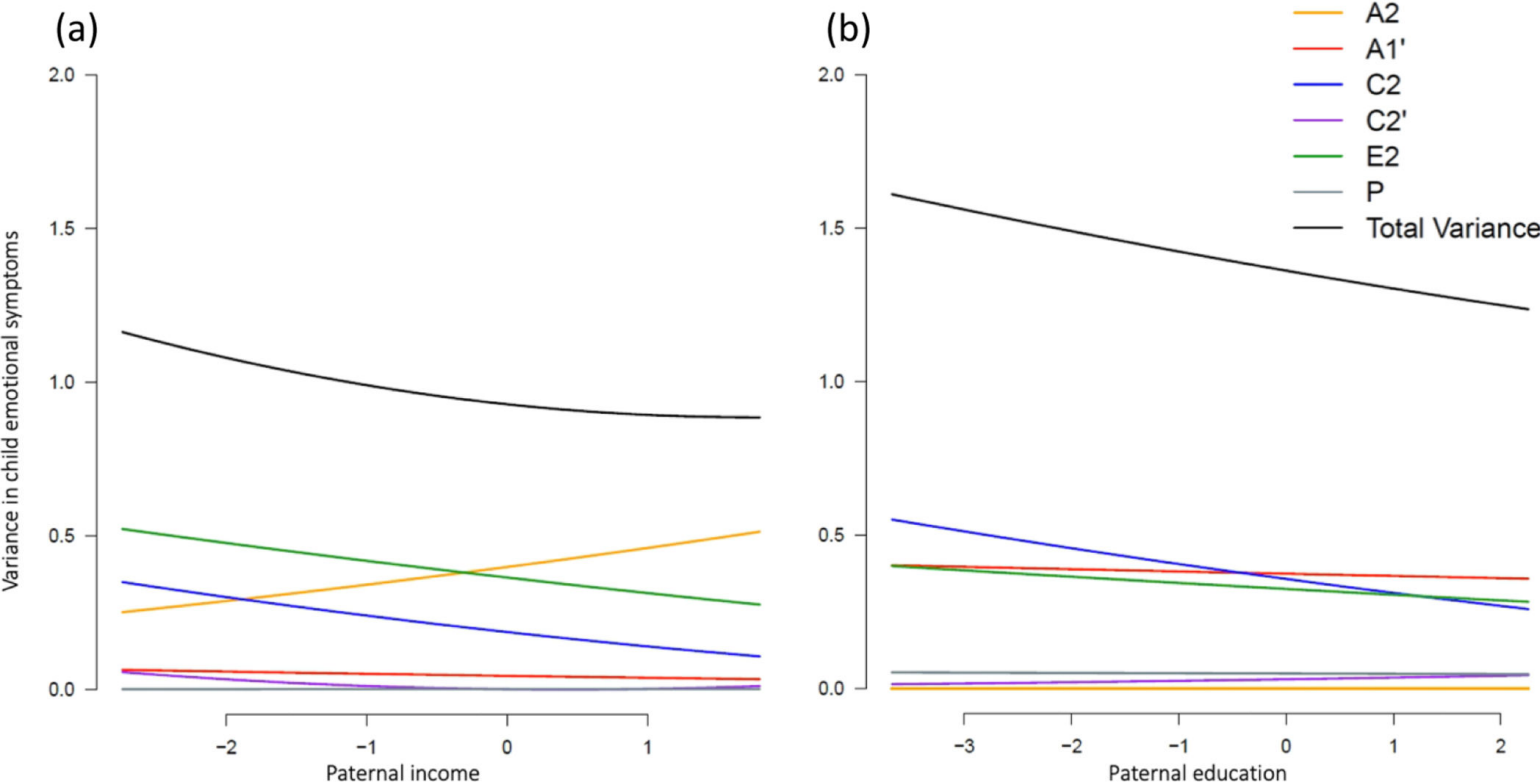
Unstandardised variance components in child emotional problems moderated by paternal income rank (a) and educational attainment (b).

Total phenotypic variance in child emotional symptoms was greater at low versus high levels of paternal income (Figure 4a) and education (Figure 4b). Figure 4a suggests that shared (C2) and non-shared environmental influences (E2) unique to child emotional outcomes were greater at lower income levels, whereas genetic influences unique to child emotional outcomes (A2) increased with increasing paternal income. Although moderation terms could not all be dropped from the model without a significant loss of model fit (Table S3), confidence intervals around the moderation terms did not highlight any single variance component as significantly moderated by paternal income (Table S5). Figure 4b suggests moderation of the shared environmental variance unique to child emotional outcomes (C2), which was greater at lower versus higher levels of paternal education. Examination of the confidence intervals around the moderation parameter estimates from the full model showed significant moderation on the shared environmental component unique to child emotional outcomes (C2) as a function of paternal education (β_yu_ = −0.05, 95% CI [−0.07, −0.02]; Table S5).

##### Behavioural symptoms

**Figure 5.**
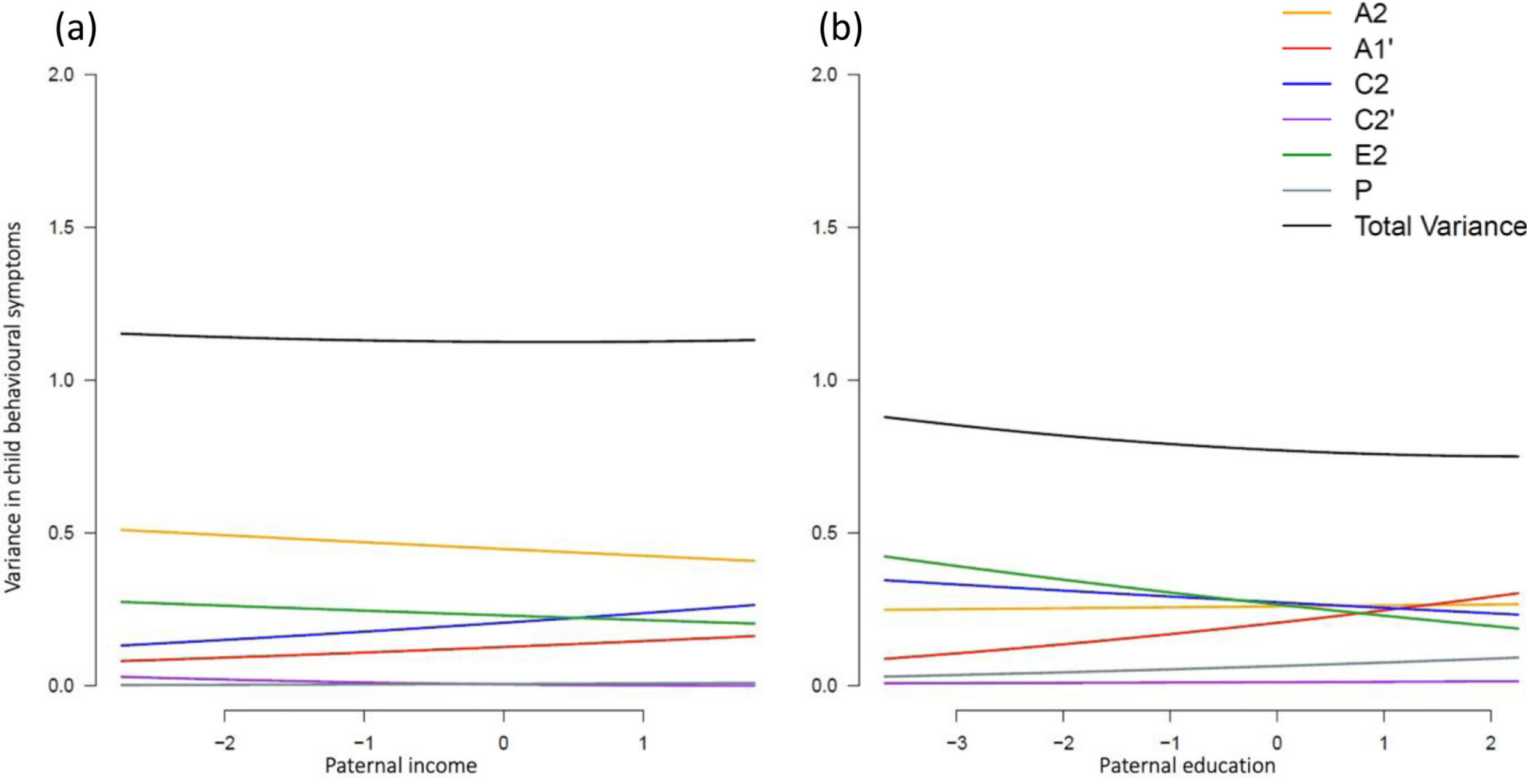
Unstandardised variance components in child behavioural problems moderated by paternal income rank (a) and educational attainment (b).

Total phenotypic variation in child behavioural symptoms was stable across levels of paternal income (Figure 5a) and education (Figure 5b). No significant moderation effects of paternal socioeconomic factors were found for child behavioural symptoms (Table S4).

## Discussion

We examined whether parental socioeconomic factors significantly moderated aetiological influences on child emotional and behavioural symptoms (GxE). In contrast to previous studies, we were able to model gene-environment correlation (rGE) and GxE simultaneously. Using extended family units, we were able to estimate the aetiological structure of socioeconomic factors and thus the genetic and environmental covariance between parents’ socioeconomic factors and child outcomes. This allowed us to investigate whether family SES moderates the influence of genetic and environmental influences unique to child emotional and behavioural problems, as well as any genetic and environmental influences shared with parental socioeconomic factors.

We found evidence that shared environmental influences unique to child emotional symptoms (C2) were higher at lower levels of maternal and paternal educational attainment. Thus, shared environmental influences explained greater variance in emotional symptoms for children in more socioeconomically disadvantaged circumstances. This is consistent with previous evidence indicating that shared environmental influences on child emotional problems have a greater influence among children from families with lower SES (Middeldorp et al., 2014). In contrast to prior studies, we did not observe significant moderation on shared environmental influences specific to child behavioural symptoms (Burt et al., 2016, 2020; Hendriks et al., 2019; Middeldorp et al., 2014; Tuvblad et al., 2006). Discrepancies could in part reflect the different measures used to index family SES. In this study, we used individual-level national register data to obtain independent information on both maternal and paternal income levels and educational attainment. In contrast, past research used either measures of neighbourhood-level disadvantage (Burt et al., 2016, 2020) or self-report data on parents’ educational attainment and occupational status, and did not distinguish paternal from maternal socioeconomic indicators (Hendriks et al., 2019; Middeldorp et al., 2014; Tuvblad et al., 2006). Reporting and response biases have been shown to affect the validity of self-report measures of SES, leading to difficulties with comparisons between studies (Angel et al., 2019; Duncan & Magnuson, 2003; Lorant et al., 2007; Moore et al., 2000). Differences between study samples, such as sample age (e.g., child outcomes assessed in early childhood versus middle childhood/adolescence) and that samples were drawn from different populations (e.g., Norway versus the USA), could also contribute to differences in the pattern of interaction effects detected.

We also found evidence that genetic influences on child behavioural symptoms that are shared with maternal income (A1’) were greater at lower levels of income. Thus, these shared genetic factors explained more of the variance in child behavioural symptoms for families at this end of the socioeconomic distribution. This finding appears to support the diathesis stress framework (Monroe & Simons, 1991), which suggests that genetic differences in behavioural outcomes manifest in poorer environments. In turn, the influence of the non-shared environment on child behavioural outcomes increased with increasing maternal income and educational attainment. This is consistent with previous evidence demonstrating that non-shared influences explained more of the variance in behavioural problems in children from families with higher parental educational attainment (Hendriks et al., 2019). Our finding that shared genetic factors explained greater variation in behavioural symptoms for children in lower SES families is in contrast with those of prior twin studies that reported lower heritability of behavioural problems for children in less advantaged environments (Burt et al., 2016; Hendriks et al., 2019; Middeldorp et al., 2014; Tuvblad et al., 2006). It is possible that previous studies have not detected increased genetic influences on child psychopathology at lower ends of the socioeconomic distribution, because the genetic effects are shared between parent SES and child psychopathology. Previous studies have regressed out this covariance and only tested for GxE on the variance that is unique to the child trait (Burt et al., 2016, 2020; Middeldorp et al., 2014; Purcell, 2002; Tuvblad et al., 2006). Our findings suggest that this may have biased findings in previous studies. In the current study, our ability to model the covariance between socioeconomic factors and child outcomes revealed that heritability was *higher* at lower levels of maternal income/education. This increased heritability was driven by genetic variance shared between maternal SES indicators and child behavioural outcomes that would have been regressed out of previous analyses. To our knowledge, ours is the first GxE study to incorporate moderation of the genetic variance that is shared between family SES and child mental health.

A possible interpretation of the finding that aetiological influences on child behavioural outcomes were moderated by maternal, but not paternal, socioeconomic indicators is that these indicators could partly be reflective of individual traits of the parents and may affect child behaviour via different pathways. The correlation between maternal and paternal income was low (*r* = .07) and given that mothers still tend to take on the majority of child caregiving duties (Astrid, 2020; Dietrichson, 2017), low income for many mothers in this sample may indicate someone who is taking on the majority of the childcare but may not be materially poor. Future work disentangling the respective contributions of different socioeconomic indicators would improve our understanding of the mechanisms underlying intergenerational associations between family SES and child mental health. While we modelled mothers and fathers separately, future analyses could also seek to model them simultaneously as an overall measure of family SES and investigate whether moderation effects differ between individual and composite measures of SES.

### Limitations

There are some limitations to our study. First, income inequality in Norway is low relative to many other countries (UNICEF, 2017), so findings from this sample may not generalise to countries with greater economic disparity. Second, participation in MoBa is characterised by self-selection. MoBa participants have been found to have a higher educational attainment and experience lower levels of mental health problems compared to those who did not participate, which may affect generalisability of our findings (R. M. Nilsen et al., 2009) However, studies suggest that reduced prevalence rates in MoBa do not necessarily lead to biases in estimates of associations between exposures and outcomes (R. M. Nilsen et al., 2009; Oerbeck et al., 2017). Third, the current results should be considered specific to early childhood. Time-specific associations between family SES and trajectories of emotional and behavioural problems have been reported (Miller et al., 2021). In addition, developmental differences in moderation could be expected given that the aetiology of mental health problems changes across development (Hannigan et al., 2017). Last, although the phenotypic associations between parental socioeconomic factors and child emotional and behavioural outcomes were in the expected direction, they were small. However, estimates were in line with those reported in previous studies (Burt et al., 2016, 2020; Tuvblad et al., 2006) and the presence of small phenotypic associations between the moderator and outcome has no bearing on the extent of aetiologic moderation (van der Sluis et al., 2012).

## Conclusion

This study uses a novel genetically informative research design in a large population-based sample. We test for gene-environment interaction (GxE) in the presence of gene-environment correlation (rGE) for environmental moderators that are necessarily shared between children growing up in the same family. We provide evidence that family socioeconomic factors moderate the influence of familial and non-shared environmental influences on child emotional and behaviour problems. This is the first study to demonstrate moderation of genetic variance that is shared between family SES and child mental health. Our findings indicate that the presence and pattern of moderation effects varies depending on the measure used to index family SES. Future studies may next consider using similar large-scale, genetically informative data to explore how the dynamics of different measures of the family socioeconomic environment relate to *trajectories* of aetiologic moderation on emotional and behavioural problems across child developmental periods.

## Supplementary Material

Subinfo

## Figures and Tables

**Table 1.** Study sample size stratified by maternal/paternal kinship

Mother-child extended families (N = 16408)		

Study sample size stratified by mothers’ relatedness	rA	n

Identical twin pair	1.0	57
Full-sibling/fraternal twin pair	.50	4963
Half-sibling pair	.25	362
Unrelated (sibling-in-law) pair	.00	11026

Number of offspring pairs linked to each mother	rA	n

Full-sibling pair	.50	4346
Maternal half-sibling pair	.25	32
Unpaired (single) offspring	---	16692

Number of offspring pairs linked to each mother in unpaired nuclear families	rA	n

Identical twin pair	1.0	167
Full-sibling/fraternal twin pair	.50	6859
Maternal half-sibling pair	.25	56

Father-child extended families (N = 16455)		

Study sample size stratified by fathers’ relatedness	rA	n

Identical twin pair	1.0	23
Full-sibling/fraternal twin pair	.50	3464
Half-sibling pair	.25	164
Unrelated (sibling-in-law) pair	.00	12804

Number of offspring pairs linked to each father	rA	n

Full-sibling pair	.50	4299
Paternal half-sibling pair	.25	16
Unpaired (single) offspring	---	16845

Number of offspring pairs linked to each father in unpaired nuclear families	rA	n

Identical twin pair	1.0	167
Full-sibling/fraternal twin pair	.50	6859
Paternal half-sibling pair	.25	0

## Key points

- Socioeconomic status (SES) has been shown to moderate aetiological influences on child mental health, however, previous gene-environment interaction (GxE) studies have not explored the potential genetic covariance between child mental health and family SES.
- We applied a novel approach using extended family data to investigate the moderation of aetiological influences on child emotional and behavioural problems by maternal and paternal socioeconomic status in the presence of gene-environment correlation.
- Family socioeconomic status indicators moderated the influence of familial and non-shared environmental influences on child emotional and behaviour problems.
- To our knowledge, this is the first study to model genetic covariance between family socioeconomic status (SES) and child mental health and to demonstrate moderation of genetic variance that is shared between family SES and child mental health.
- Our findings suggest that family SES largely influences child emotional and behavioural problems indirectly via interaction with genetic and environmental factors influencing susceptibility and resilience.

## Footnotes

1 We did not fit constrained sub models to test for the significance of individual moderated parameters because omission of moderation effects by fixing them to 0 can bias estimation of parameters (e.g., dropping ßc can inflate ßa, and there are issues of specificity in distinguishing between ßa and ßc; see Figure S2) (Van Hulle & Rathouz, 2015).
