# Supplementary material for: Socioeconomic status and risk for child psychopathology: Exploring gene-environment interaction in the presence of gene-environment correlation using extended families in the Norwegian Mother, Father and Child Birth Cohort Study
Source: J Child Psychol Psychiatry. Author manuscript; Available in PMC 2025 Feb 1. (PMC10859652; doi:10.1111/jcpp.13872)
Supplement: Subinfo [file NIHMS1920193-supplement-Subinfo.docx]

**Supporting Information**

# Table S1. Phenotypic correlations between all study variables.

| Variable |  | *M* | *SD* | 1 | 2 | 3 | 4 | 5 | 6 | 7 | 8 | 9 | 10 |
| --- | --- | --- | --- | --- | --- | --- | --- | --- | --- | --- | --- | --- | --- |
| 1.Maternal age | | 30.30 | 4.49 |  |  |  |  |  |  |  |  |  |  |
| 2. Paternal age | | 32.28 | 5.26 | .67** |  |  |  |  |  |  |  |  |  |
|  | |  |  | [.66, .67] |  |  |  |  |  |  |  |  |  |
| 3. Parity | | 0.78 | 0.88 | .40** | .32** |  |  |  |  |  |  |  |  |
|  | |  |  | [.40, .41] | [.31, .32] |  |  |  |  |  |  |  |  |
| 4. Child year of | | 2005.07 | 2.20 | .07** | .05** | -.07** |  |  |  |  |  |  |  |
| birth | |  |  | [.06, .08] | [.04, .06] | [-.08, -.07] |  |  |  |  |  |  |  |
| 5. Child sex | | 1.49 | 0.50 | .00 | -.00 | -.00 | -.00 |  |  |  |  |  |  |
|  | |  |  | [-.01, .01] | [-.01, .01] | [-.01, .01] | [-.01, .01] |  |  |  |  |  |  |
| 6. Child emotional | | 1.25 | 0.20 | -.08** | -.04** | -.08** | -.02** | .03** |  |  |  |  |  |
| problems | |  |  | [-.09, -.08] | [-.05, -.03] | [-.08, -.07] | [-.03, -.02] | [.02, .04] |  |  |  |  |  |
| 7. Child behavioural | | 1.47 | 0.26 | -.09** | -.05** | -.06** | -.08** | -.08** | .39** |  |  |  |  |
| problems | |  |  | [-.09, -.08] | [-.05, -.04] | [-.07, -.05] | [-.09, -.08] | [-.08, -.07] | [.38, .39] |  |  |  |  |
| 8. Maternal registry | | 0.57 | 0.24 | .06** | .02** | -.10** | .09** | .01 | -.08** | -.05** |  |  |  |
| income | |  |  | [.06, .07] | [.01, .02] | [-.10, -.09] | [.08, .10] | [-.00, .01] | [-.09, -.08] | [-.05, -.04] |  |  |  |
| 9. Maternal registry | | 5.37 | 1.38 | .22** | .11** | -.09** | .15** | .01 | -.14** | -.14** | .38** |  |  |
| education | |  |  | [.21, .22] | [.10, .12] | [-.10, -.09] | [.14, .16] | [-.00, .01] | [-.14, -.13] | [-.14, -.13] | [.37, .38] |  |  |
| 10. Paternal registry | | 0.61 | 0.23 | .05** | -.04** | -.01** | .04** | .00 | -.06** | -.05** | .07** | .16** |  |
| income | |  |  | [.05, .06] | [-.04, -.03] | [-.02, -.00] | [.03, .05] | [-.01, .01] | [-.06, -.05] | [-.06, -.05] | [.06, .08] | [.15, .17] |  |
| 11. Paternal registry | | 5.06 | 1.54 | .20** | .08** | -.03** | .10** | .00 | -.10** | -.11** | .19** | .45** | .33** |
| education | |  |  | [.19, .21] | [.07, .09] | [-.03, -.02] | [.10, .11] | [-.01, .01] | [-.11, -.09] | [-.11, -.10] | [.18, .20] | [.44, .46] | [.33, .34] |

*Note.* *M* and *SD* are used to represent mean and standard deviation, respectively. Values in square brackets indicate the 95% confidence interval for each correlation. * indicates *p* < .05. ** indicates *p* < .01.

# Table S2. Fit statistics from the biometric moderation MCoTS models of child emotional and behavioural outcomes moderated by maternal SES indices.

|  | ∆ -2LL | ∆ *df* | *p* | AIC |
| --- | --- | --- | --- | --- |
| **Maternal income and child emotional problems** | | | | |
| Full moderation | - | - | - | 188201.00 |
| No moderation | 85.78 | 6 | 2.28e-16 | 188274.80 |
| **Maternal education and child emotional problems** | | | | |
| Full moderation |  |  |  | 175788.00 |
| No moderation | 155.74 | 6 | 4.73e-31 | 175931.70 |
| **Maternal income and child behavioural problems** | | | | |
| Full moderation | - | - | - | 188551.30 |
| No moderation | 12.57 | 6 | 0.05 | 188551.90 |
| **Maternal education and child behavioural problems** | | | | |
| Full moderation | - | - | - | 176167.40 |
| No moderation | 45.32 | 6 | 4.05e-08 | 176200.70 |
| -2LL = -2 log likelihood; df = degrees of freedom; AIC = Akaike’s Information Criterion. | | | | |

# Table S3. Moderated path estimates, standard errors and 95% confidence intervals of child emotional and behavioural outcomes moderated by maternal SES indices.

| Child emotional problems | | | | | | | | | |
| --- | --- | --- | --- | --- | --- | --- | --- | --- | --- |
|  | Maternal income | | |  |  | Maternal education | | | |
| Parameter | Estimate | Std. error | LL | UL |  | Estimate | Std. error | LL | UL |
| β_xu_ | -0.06 | 0.06 | -0.14 | 0.14 |  | 0.11 | 0.04 | -0.17 | 0.17 |
| β_yu_ | -0.03 | 0.03 | -0.08 | 0.03 |  | **-0.06** | **0.02** | **-0.11** | **-0.01** |
| β_xc_ | 0.02 | 0.05 | -0.07 | 0.11 |  | 0.05 | 0.04 | -0.04 | 0.12 |
| β_zu_ | 0.01 | 0.03 | -0.05 | 0.06 |  | 0.01 | 0.02 | -0.03 | 0.05 |
| β_yc_ | -0.09 | 0.03 | -0.14 | 0.14 |  | -0.04 | 0.07 | -0.16 | 0.16 |
| β_zc_ | -0.01 | 0.02 | -0.04 | 0.03 |  | -0.03 | 0.02 | -0.06 | 0.01 |

| Child behavioural problems | | | | | | | | | |
| --- | --- | --- | --- | --- | --- | --- | --- | --- | --- |
|  | Maternal income | | |  |  | Maternal education | | | |
| Parameter | Estimate | Std. error | LL | UL |  | Estimate | Std. error | LL | UL |
| β_xu_ | 0.00 | 0.05 | -0.09 | 0.09 |  | 0.00 | 0.23 | -0.17 | 0.17 |
| β_yu_ | 0.00 | 0.01 | -0.03 | 0.04 |  | -0.03 | 0.07 | -0.08 | 0.05 |
| β_xc_ | **0.06** | **0.02** | **0.01** | **0.09** |  | 0.08 | 0.14 | -0.03 | 0.14 |
| β_zu_ | **0.06** | **0.02** | **0.02** | **0.09** |  | **0.06** | **0.03** | **0.02** | **0.08** |
| β_yc_ | 0.02 | 0.03 | -0.03 | 0.08 |  | 0.09 | 0.06 | -0.15 | 0.15 |
| β_zc_ | -0.01 | 0.01 | -0.03 | 0.01 |  | -0.04 | 0.04 | -0.07 | 0.01 |

*Note:* Moderated components estimated under the full moderation model. The parameter estimates for the mean and unmoderated parameters are not shown. β_xu_, β_yu_ and β_zu_ = moderated components of A2, C2 and E2 (i.e. variance components unique to child emotional or behavioural traits). β_xc_, β_yc_ and β_zc_ = moderated components of A1’, C1’ and p (i.e. variance components common to parent SES and child emotional or behavioural traits). LL = lower bound of the 95% confidence interval. UL = upper bound of the 95% confidence interval.

# Table S4. Fit statistics from the biometric moderation MCoTS models of child emotional and behavioural outcomes moderated by paternal SES indices.

|  | | | | |
| --- | --- | --- | --- | --- |
|  | ∆ -2LL | ∆ *df* | *p* | AIC |
| **Paternal income and child emotional problems** | | | | |
| Full moderation | - | - | - | 185850.90 |
| No moderation | 59.98 | 6 | 4.55e-11 | 185898.90 |
| **Paternal education and child emotional problems** | | | | |
| Full moderation | - | - | - | 170848.20 |
| No moderation | 83.53 | 6 | 6.64e-16 | 170919.80 |
| **Paternal income and child behavioural problems** | | | | |
| Full moderation | - | - | - | 186114.00 |
| No moderation | 4.38 | 6 | 0.63 | 186106.40 |
| **Paternal education and child behavioural problems** | | | | |
| Full moderation | - | - | - | 171142.60 |
| No moderation | 10.01 | 6 | 0.12 | 171140.60 |
| -2LL = -2 log likelihood; df = degrees of freedom; AIC = Akaike’s Information Criterion. | | | | |

# Table S5. Moderated path estimates, standard errors and 95% confidence intervals of child emotional and behavioural outcomes moderated by paternal SES indices.

| Child emotional problems | | | | | | | | | |
| --- | --- | --- | --- | --- | --- | --- | --- | --- | --- |
|  | Paternal income | | |  |  | Paternal education | | | |
| Parameter | Estimate | Std. error | LL | UL |  | Estimate | Std. error | LL | UL |
| β_xu_ | 0.04 | 0.05 | -0.13 | 0.13 |  | 0.00 | 0.08 | -0.10 | 0.10 |
| β_yu_ | -0.06 | 0.04 | -0.12 | 0.07 |  | **-0.04** | **0.01** | **-0.06** | **-0.02** |
| β_xc_ | 0.03 | 0.09 | -0.12 | 0.14 |  | 0.01 | 0.03 | -0.06 | 0.07 |
| β_zu_ | -0.04 | 0.03 | -0.08 | 0.04 |  | -0.02 | 0.03 | -0.06 | 0.03 |
| β_yc_ | -0.08 | 0.06 | -0.15 | 0.15 |  | 0.01 | 0.04 | -0.07 | 0.08 |
| β_zc_ | 0.00 | 0.03 | -0.05 | 0.05 |  | 0.00 | 0.02 | -0.04 | 0.03 |
| Child behavioural problems | | | | | | | | | |
|  | Paternal income | | |  |  | Paternal education | | | |
| Parameter | Estimate | Std. error | LL | UL |  | Estimate | Std. error | LL | UL |
| β_xu_ | -0.02 | 0.03 | -0.08 | 0.06 |  | 0.00 | 0.06 | -0.10 | 0.10 |
| β_yu_ | 0.03 | 0.02 | -0.02 | 0.08 |  | -0.01 | 0.03 | -0.06 | 0.03 |
| β_xc_ | -0.03 | 0.04 | -0.10 | 0.05 |  | 0.04 | 0.04 | -0.06 | 0.11 |
| β_zu_ | -0.02 | 0.02 | -0.06 | 0.02 |  | -0.04 | 0.02 | NA | 0.02 |
| β_yc_ | -0.04 | 0.04 | -0.10 | 0.10 |  | 0.00 | 0.03 | -0.06 | 0.07 |
| β_zc_ | 0.01 | 0.01 | -0.02 | 0.04 |  | -0.02 | 0.02 | NA | 0.03 |

*Note:* Moderated components estimated under the full moderation model. The parameter estimates for the mean and unmoderated parameters are not shown. β_xu_, β_yu_ and β_zu_ = moderated components of A2, C2 and E2 (i.e. variance components unique to child emotional or behavioural traits). β_xc_, β_yc_ and β_zc_ = moderated components of A1’, C1’ and p (i.e. variance components common to parent SES and child emotional or behavioural traits). LL = lower bound of the 95% confidence interval. UL = upper bound of the 95% confidence interval.

# Figure S1. Path diagram of the full Multiple-Children-of-Twins-and-Siblings (MCoTS) structural equation model.


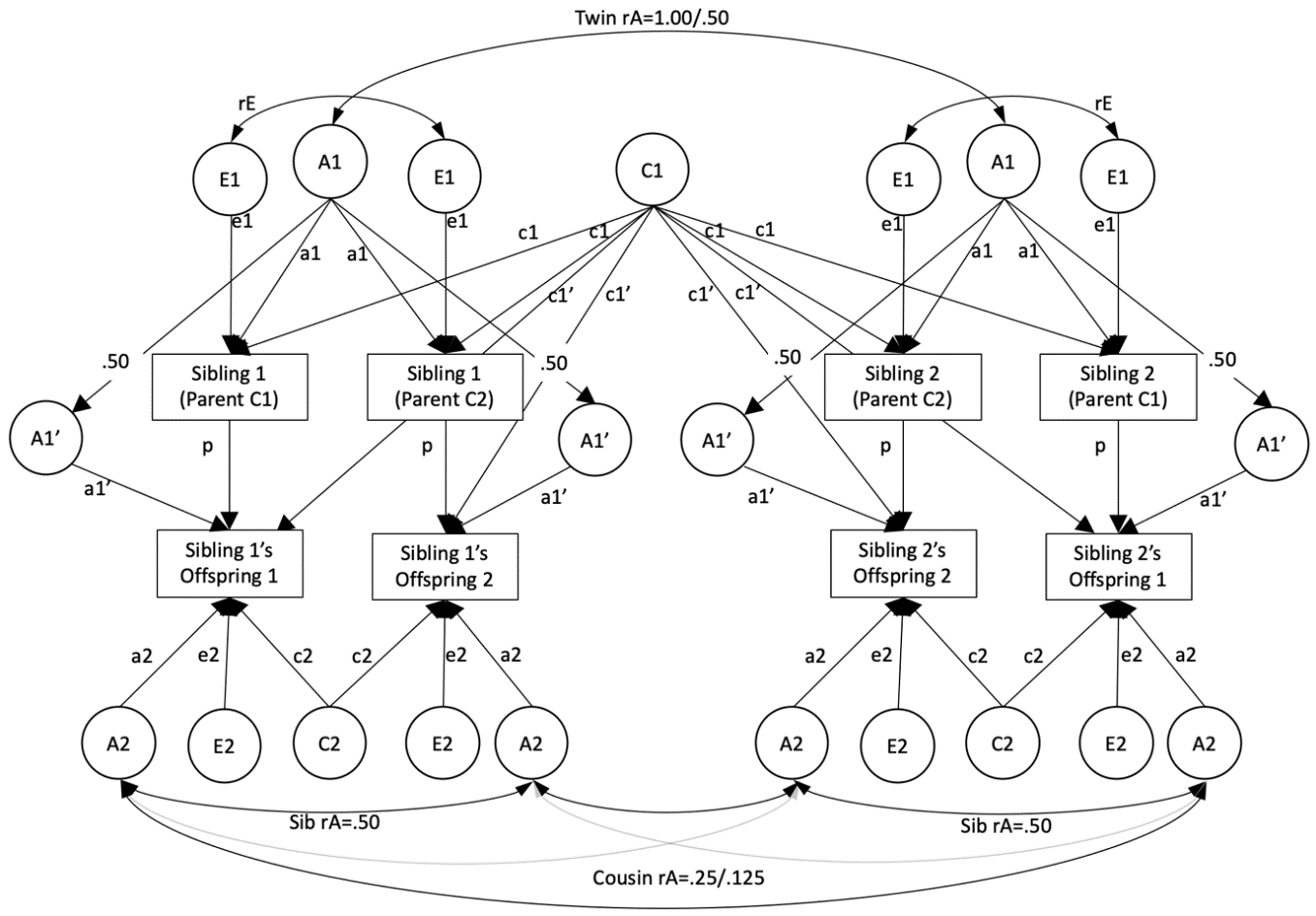


*Note*. The parent trait varies across offspring. A1 = additive genetic effects on parental trait; C1 = shared environmental effects on parental trait; E1 = nonshared environmental effects on parental trait; A1’ = genetic effects shared between parental trait and offspring trait; C1’ = extended family effects (i.e. shared environment of the parents influences offspring trait); A2 = genetic effects specific to offspring trait; C2 = shared environmental effects on offspring trait; E2 = nonshared environmental effects on offspring trait; p = residual phenotypic association after accounting for genetic and environmental overlap; rE = within-parent correlation between E1 for parent trait of child 1 and 2. The path between A1 and A1’ is fixed to 0.5 because parents share half their DNA with their children.

# Figure S2. Purcell (2002) bivariate moderation model.


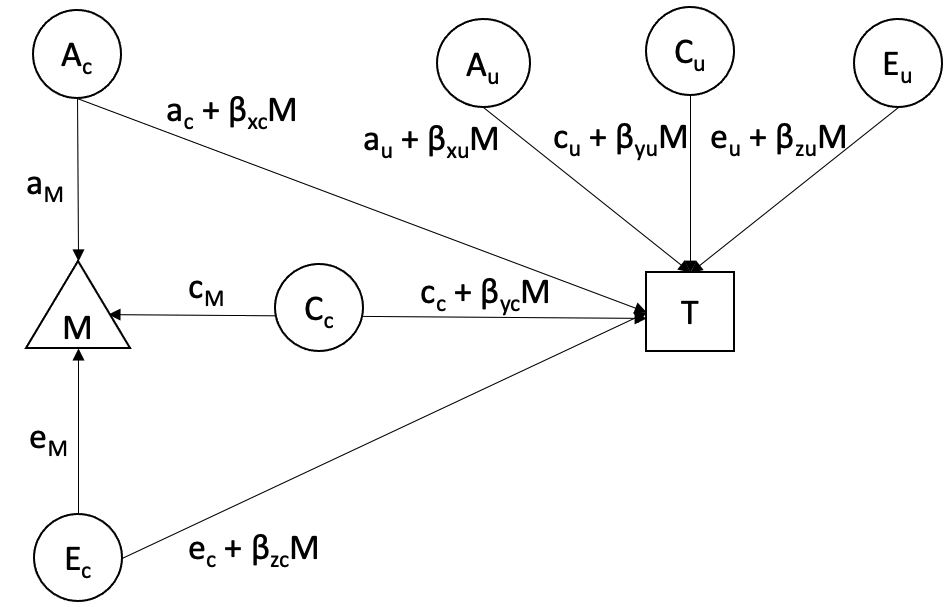


*Note:* Bivariate moderation model shown for only one member of a twin pair as proposed by Purcell (2002). Ac, Cc and Ec are the variance components common to the moderator and the trait. Au, Cu, and Eu are the variance components unique to the trait. β coefficients index the direction and magnitude of moderation. The total variance of the trait can be calculated as follows: Var(T|M) = (a_c_ + β_xc_M)^2^ + (a_u_ + β_xu_M)^2^ + (c_c_ + β_yc_M)^2^ + (c_u_ + β_yu_M)^2^ + (e_c_ + β_zc_M)^2^ + (e_u_ + β_zu_M)^2^.

# Figure S3. Distributions of child emotional and behavioural outcome variables before and after logarithmic transformation.

**(a)**


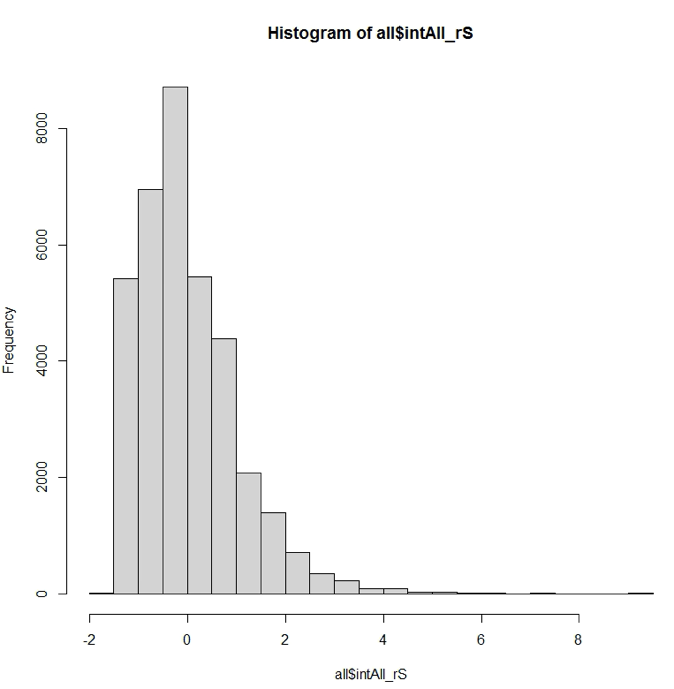

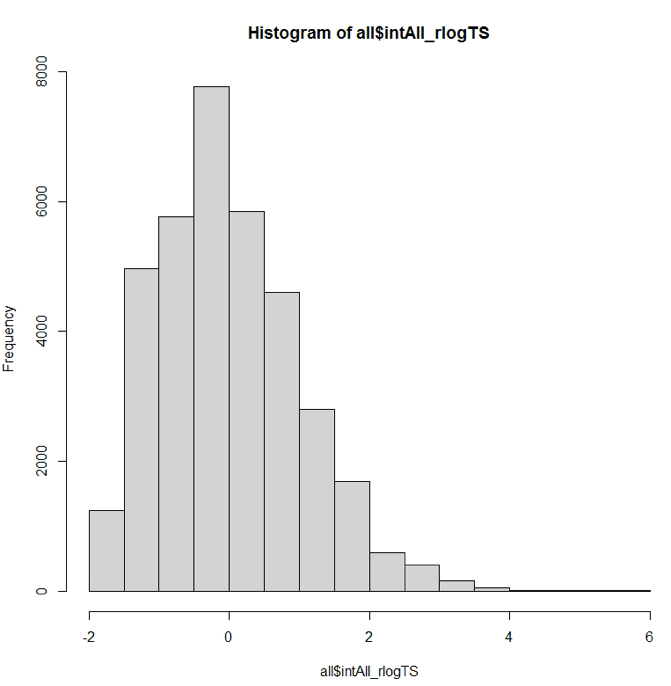


Skew = 1.28

Kurtosis = 3.05

Skew = 0.64

Kurtosis = 0.41

**(b)**
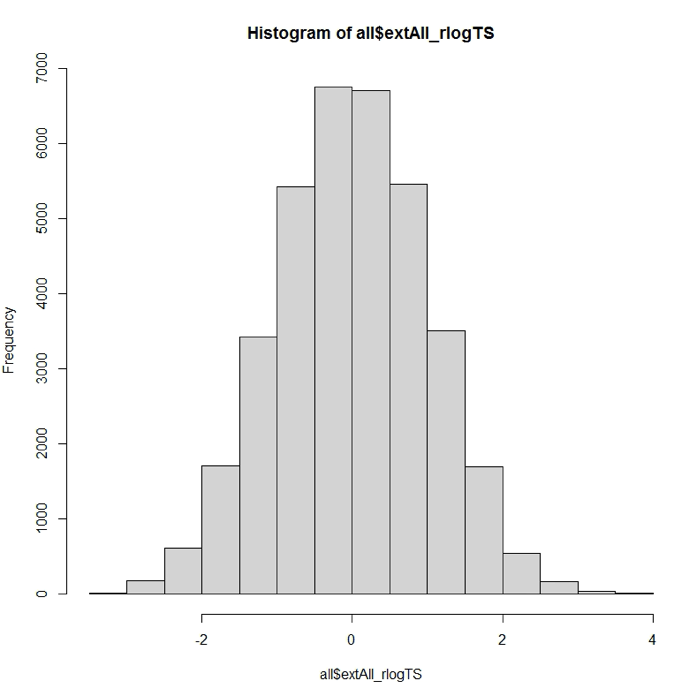

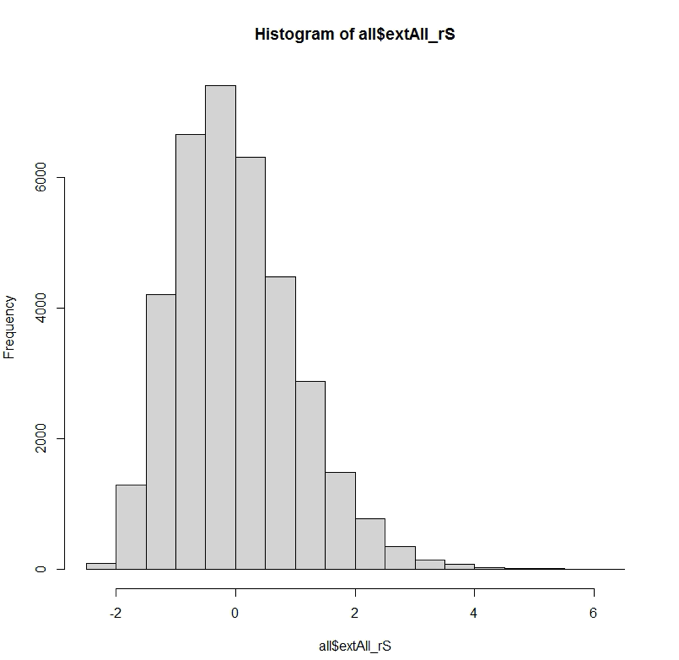


Skew = 0.67

Kurtosis = 0.63

Skew = 0

Kurtosis = -0.21

*Note:* **(a)** Histograms of emotional scores scale before (left) and after log transformation (right). **(b)** Histograms of behavioural scores scale before (left) and after log transformation (right).
